# The Effects of Arbuscular Mycorrhizal Fungal Colonisation on Nutrient Status, Growth, Productivity, and Canker Resistance of Apple (*Malus pumila*)

**DOI:** 10.3389/fmicb.2018.01461

**Published:** 2018-07-03

**Authors:** Despina Berdeni, T. E. A. Cotton, Tim J. Daniell, Martin I. Bidartondo, Duncan D. Cameron, Karl L. Evans

**Affiliations:** ^1^Department of Animal and Plant Sciences, University of Sheffield, Sheffield, United Kingdom; ^2^Ecological Sciences, The James Hutton Institute, Dundee, United Kingdom; ^3^Department of Life Sciences, Imperial College London, London, United Kingdom; ^4^Comparative Plant and Fungal Biology, Royal Botanic Gardens, Kew, Richmond, United Kingdom

**Keywords:** arbuscular mycorrhizal fungi, apple canker, biotic resistance, nutrient status, pathogens, root growth, symbiosis, sustainable agriculture

## Abstract

We assess whether arbuscular mycorrhizal fungi (AMF) improve growth, nutritional status, phenology, flower and fruit production, and disease resistance in woody perennial crops using apple (*Malus pumila)* as a study system. In a fully factorial experiment, young trees were grown for 3 years with or without AMF (*Funneliformis mosseae* and *Rhizophagus irregularis*), and with industrial standard fertiliser applications or restricted fertiliser (10% of standard). We use two commercial scions (Dabinett and Michelin) and rootstocks (MM111 and MM106). Industrial standard fertiliser applications reduced AMF colonisation and root biomass, potentially increasing drought sensitivity. Mycorrhizal status was influenced by above ground genotypes (scion type) but not rootstocks, indicating strong interactions between above and below ground plant tissue. The AMF inoculation significantly increased resistance to *Neonectria ditissima*, a globally economically significant fungal pathogen of apple orchards, but did not consistently alter leaf nutrients, growth, phenology or fruit and flower production. This study significantly advances understanding of AMF benefits to woody perennial crops, especially increased disease resistance which we show is not due to improved tree nutrition or drought alleviation. Breeding programmes and standard management practises can limit the potential for these benefits.

## Introduction

Increased use of inorganic fertilisers has made a huge contribution to growth in agricultural yields in recent decades and agricultural production must continue to expand to meet growing food demands (Pretty et al., 2010; Tilman et al., 2011). One of the biggest challenges to sustainable global food security is the need to find suitable replacements for inorganic fertilisers. This is because inorganic fertiliser production consumes an increasing proportion of the global energy budget and the supply of key raw materials, primarily phosphorus, is becoming limited (Herrera-Estrella and López-Arredondo, 2016), causing price increases that reduce the availability of fertilisers for poorer farmers and increase food costs (Cordell et al., 2009; Cordell and White, 2014). In addition, inorganic fertilisers have significantly contributed to pollution of water and the atmosphere (Elser and Bennett, 2011; Tubiello et al., 2015; Bauer et al., 2016) and reduced terrestrial biodiversity due to habitat degradation and loss (Tilman et al., 2017). To prevent further environmental damage and ensure food security for future generations it is therefore imperative to find strategies to balance productivity with environmental sustainability (Foley et al., 2011; Horton, 2017).

Arbuscular mycorrhizal fungi (AMF) are able to form symbioses with the majority of plant species including many important crops (Öpik et al., 2006; Smith and Read, 2008). The AMF are recognised as an integral component of agro-ecosystems (Thirkell et al., 2017). In exchange for carbon, AMF provide plants with essential nutrients – most notably phosphorus – which they efficiently forage from the soil via extensive hyphal networks, thus potentially reducing the need for inorganic fertiliser (Fester and Sawers, 2011). In addition to nutrient provision, AMF may also directly benefit crop species through increased resistance to disease (Jung et al., 2012), tolerance to drought and adverse soil conditions (Augé, 2004; Daei et al., 2009), competitive ability over non-mycorrhizal plants (Cameron, 2010; Veiga et al., 2011) and indirectly through improved soil structure (Rillig and Mummey, 2006) and increased soil nutrient retention (Bender et al., 2015; Cavagnaro et al., 2015; Köhl and van der Heijden, 2016).

Despite potential benefits, many factors can limit mycorrhizas and thus their use in modern agricultural systems, including physical disturbance of hyphal networks through tillage (Bowles et al., 2017b), high application of inorganic fertilisers and fungicides (Mader et al., 2002; Wilson et al., 2009) and selective breeding of modern crop varieties which may inadvertently reduce the capacity of plants to form an effective AMF symbiosis (Pérez-Jaramillo et al., 2016; Leff et al., 2017).

Research on interactions between crop plants and mycorrhizal fungi has focused on arable crops, yet many woody perennial crops (e.g., grapes, *Vitis vinifera*, raspberries, *Rubus idaeus*, and currants, *Ribes* sp.), and a wide range of fruit and nut bearing trees can also form symbioses with AMF. These products contain a wide range of vitamins and micronutrients and play a major role in delivering nutritional food security (Joosten et al., 2015). Woody perennial crops also contribute significantly to global agricultural production, for example the domestic apple (*Malus pumila*) is the fourth most widely cultivated fruit crop worldwide with an estimated 89 million tonnes grown annually on over five million hectares in 2016 and is of considerable economic importance (FAO, 2018).

Disease is a major threat to the establishment and persistence of apple orchards (McCracken et al., 2003). Apple canker caused by the fungal pathogen *Neonectria ditissima* is the second most important pathogen in economic terms, causing up to 30% loss of orchard yields (Weber, 2014). It is particularly problematic in warm and wet regions of Europe (Beresford and Kim, 2010) where current control methods require regular and intensive fungicide applications combined with removal of infected material imposing direct economic costs and reduced yields (Cooke, 1999; Garkava-Gustavsson et al., 2013).

Initial work provides evidence that inoculation of young apple trees with AMF may reduce incidence of the fungal pathogens *Dematophora necatrix* and *Botryosphaeria* sp. (Raj and Sharma, 2009; Krishna et al., 2010) and improve seedling growth rates and nutritional status (Miller et al., 1985; An et al., 1993; Matsubara et al., 1996; Forge et al., 2001); however, these studies are of limited relevance to apple growers due to their short duration and as commercial apple trees are never grown from seedlings, but propagated clonally as grafted rootstocks and scions. Indeed, the extent to which interactions between the rootstock and scion, and their genetic identities, determines the magnitude of AMF induced benefits is almost entirely unknown (Albacete et al., 2015). To our knowledge no research to date has assessed how AMF influence apple resistance to *N. ditissima* or how AMF may influence important aspects of apple productivity including flower production, phenology and subsequent yield despite growing evidence that AMF may affect flower number, size, and quality (Gange et al., 2005; Varga and Kytöviita, 2010), and phenology (Vaingankar and Rodrigues, 2015; Liu et al., 2017) in other crop species.

Here, we use a unique fully factorial experiment conducted over 3 years to quantify how mycorrhizas influence the performance of two of the most widely planted apple rootstocks (MM106 and MM111) and cider apple scion varieties (Dabinett and Michelin). Our treatments establish mycorrhizal and non-mycorrhizal control apple trees, grown with industrial standard inorganic fertiliser applications recommended for commercial growers (‘high’ nutrient) and reduced fertiliser conditions (‘low’ nutrient). Specifically we tested the hypotheses that mycorrhizas positively influence (i) tree nutrient status (leaf tissue nitrogen, phosphorus, carbon, carbon:nitrogen ratio, and chlorophyll content), (ii) tree growth (height, trunk diameter, above and below ground biomass, root length, leaf and flower phenology), (iii) productivity (flower and fruit production), and (iv) resistance to the pathogen *N. ditissima*. In doing so, we also test the hypotheses that benefits of mycorrhizas are greater for trees receiving low fertiliser inputs; and that rootstock and scion types would affect the symbiosis of the trees with mycorrhizas and resultant benefits. In combination these analyses provide the first robust assessment of AMF on multiple performance indicators of a woody perennial crop in an agriculturally relevant context.

## Materials and Methods

### Experimental Design

In February 2013 a fully factorial experimental design was established using four treatment levels (i) mycorrhizal inoculum (addition or no addition), (ii) fertiliser (high or low input), (iii) scion type (Dabinett or Michelin), (iv) rootstock type (MM111 or MM106). This amounted to a total of 16 treatments replicated 10 times to give a total of 160 trees. To ensure even distribution of trees from all treatments across the experimental orchard and thus reduce potential bias from any microclimate differences, a grid layout and block design was established. Trees were grown in pots positioned in a grid comprised of 20 rows of eight trees with 1.6 m spacing between rows and 1.2 m within rows. The grid was split into 10 pairs of adjacent rows or ‘blocks.’ Each block contained one tree from each treatment and within block positions were randomly assigned. One-year-old apple trees (*Malus pumila*) were planted in 80 L volume plastic pots (55 cm diameter, 40 cm depth). Pots were filled with sharp sand (0.2–0.3 mm grade) and pure peat homogenised 1:1 by volume and adjusted to pH 7 by addition of lime. These substrates were selected due to their naturally low levels of AMF inoculum. A 3 cm deep layer of black polyethylene beads (3.5 mm diameter) was added to the surface of each pot to reduce the interaction between substrate and environment and minimise drying. The experiment was established at the Arthur Willis Environment Centre, Sheffield, United Kingdom in January 2013 (N 53°22’51” W 1°29’58”) and monitored over three consecutive growing seasons (2013–2015).

#### Mycorrhizal Inoculum

All trees within the mycorrhizal treatment were inoculated with a mixed inoculum containing both *Funneliformis mosseae* and *Rhizophagus irregularis* spores and colonised root fragments (Plantworks Ltd., United Kingdom). According to supplier instructions, 400 ml of inoculum was mixed with 9 g polyacrylamide gel powder and 1 l distilled water to form a thick paste and applied to bare tree roots immediately before planting. Trees which were not inoculated with AMF received gel and distilled water only. To prevent contamination with AMF, before planting tree root systems were thoroughly washed and all soil and all fine roots (in which mycorrhizal associations typically form) were removed. We did not autoclave the growing medium as we did not wish to grow trees in conditions in which all microbes other than AMF were absent as vegetation does not naturally grow under such conditions. Subsequent root sampling and quantification of colonisation confirmed that this treatment was effective and that the non-inoculated trees remained free of AMF colonisation throughout the course of this study (see section “Mycorrhizal Colonisation”).

#### Nutrient Treatment

Nutrient treatments were based on the DEFRA recommendations for a newly planted cider apple orchard (DEFRA, 2010) (**Supplementary Notes S1**). Trees in the ‘High’ nutrient treatment received the recommended amount of N, P, K, Mg whilst ‘Low’ nutrient trees received 10% of the recommended amounts (**Supplementary Tables S1**, **S2**). Nutrient applications were applied, in solution, fortnightly over a 20-week period during the growing season beginning from bud burst. Supplementary water was provided equally to all trees to prevent water stress.

#### Mycorrhizal Colonisation

To quantify mycorrhizal colonisation, root sampling was conducted during the final growing season and before leaf drop (September 2015) with three soils cores removed beneath each tree (corer diameter 4.5 cm, length 20.5 cm). Roots were carefully removed and washed with distilled water before fine roots (<1 mm diameter) were pooled per tree and cut into 1 cm sections. Mycorrhizal colonisation of roots was visualised using the staining technique of Brundrett et al. (1994) with the following adaptations; 15 cm of fine root per tree was cleared in 10% (w/v) KOH (80°C, 4.5 h), rinsed in distilled water and acidified with 10% (v/v) HCl (10 min). Roots were then stained in Trypan Blue (20 min) before de-staining in 50% (v/v) glycerol (30 min). Percentage root colonisation by mycorrhizal fungi was quantified using a magnified intersection method following McGonigle et al. (1990) whereby presence/absence of fungal hyphae, arbuscules, or vesicles were recorded at 100 randomly selected locations along 15 cm of fine root per tree.

#### Leaf Nutrient Status

To quantify tree nutrient status five newly developed leaves were randomly collected per tree during July (i.e., mid growing season) of each experimental year (2013, 2014, and 2015). Leaves were oven dried (80°C, 48 h) before being homogenised. To quantify total phosphorus, a 25 mg subsample was digested in sulphuric acid and hydrogen peroxide following Allen (1989). After dilution (*N* = 1:10 distilled water) Murphy–Riley colorimetric P-determination (Allen, 1989) was performed at 882 nm using a Cecil Ce 1020 spectrophotometer (Cecil Instruments Ltd., United Kingdom). Total nitrogen and carbon was measured for homogenised leaf tissue subsamples of *c*. 5 mg per tree using an elemental analyser (VarioEl Cube; Isoprime, Germany). Leaf tissue carbon and C:N ratios were measured as both are useful indicators of plant nutrition and investment in defence (Royer et al., 2013).

#### Leaf Chlorophyll Content

As a physiological indicator of health, leaf chlorophyll was measured monthly over the growing season (April – September) during 2013–2015. At each time point mean leaf chlorophyll content per tree was quantified by measurement of five leaves per tree using a portable SPAD-502 chlorophyll meter (Minolta Camera Ltd., Japan). To calculate chlorophyll (mg g^−1^ dry leaf tissue) values for corresponding chlorophyll meter readings, we used ice cold acetone extraction and quantification of chlorophyll for a subsample of leaves following Cameron et al. (2009). Twenty chlorophyll readings were recorded per leaf for one leaf from each of 12 apple trees (three trees per scion/rootstock combination). Leaves were harvested and kept on ice in the dark before chlorophyll extraction from a 25–50 mg subsample per leaf (within 1 h). The optical density of the supernatant was then measured at 645 and 663 nm using a Cecil Ce 1020 spectrophotometer (Cecil Instruments Ltd., United Kingdom). A further subsample of fresh material per leaf was oven dried (80°C, 48 h) to allow chlorophyll quantification per gram of dry tissue weight. Total chlorophyll concentration (mg l^−1^) was calculated according to Arnon (1949) and expressed as mg chlorophyll per gram of dry leaf tissue.

#### Tree Growth

To quantify tree growth, height and trunk diameter measurements were recorded at the beginning of the growing season (before bud burst) and following leaf senescence in autumn. Height was recorded to the nearest cm from the base of the trunk vertically to the tip of the highest branch. Trunk diameter (mm) was measured 20 cm from the base of the trunk. To account for any irregularities in trunk shape, diameter was calculated as the average of two diameter measures taken perpendicular from the trunk centre.

#### Biomass

Destructive biomass harvest was conducted for three randomly selected blocks of trees (three replicates per treatment, 48 trees in total) in December 2015 after three growing seasons (34 months). Trees were removed from pots intact and root systems were thoroughly washed. Tree biomass was separated into above and below ground material and fresh weights recorded. Above ground biomass was chipped and a subsample of *c.* 40 g was weighed then oven dried at 98°C to a constant dry mass. Total dry above ground biomass per tree was calculated based on subsample water content. Roots were air dried at room temperature (fine roots are too fragile for oven drying) to a constant mass.

#### Root Growth

Root growth was measured using mini-rhizotron imaging. Immediately after planting, clear acrylic mini-rhizon tubes (50 cm length × 7 cm diameter) sealed at the lower end, were installed in each pot at 45° to the soil substrate surface. The above ground section of each tube was painted black to exclude light, then white to reduce heat absorption and sealed with a white cap to protect from precipitation. At the end of each growing season (October) mini-rhizotron tubes were scanned fully (two images per tube, image size 19.55 cm × 21.57 cm, resolution 400 dpi) using a CID-600 roots scanner (CID, Kansas, United States). For each root scan, root length was measured by manual digitalisation of all visible roots followed by line length measurement using ImageJ software (Schneider et al., 2012).

#### Fruit Production

Fruit production was low for all experimental years due to the immaturity of the trees. Fruit number and fresh biomass per tree were recorded at the end of the first growing season (2013); however, due to canker infection fruit yield was especially low in 2014 and 2015. Therefore for these years total flower number per tree was recorded as an indicator of potential apple yield.

#### Leaf and Flower Phenology

Leaf phenology was recorded during year two (2014) and year three (2015) from the 1st March. At *c.* 5-day intervals (range 4–6 days) the number of leaf buds to have reached each of the following developmental stages was estimated. Developmental stages were defined as (i) leaf bud dormant, (ii) bud swollen – heavily swollen but no sign of opening, (iii) bud beginning to open but less than half is green, (iv) over half of the bud is green but leaf tips point inward, (v) leaf tips point outward and leaf unfurling is clear, (vi) leaves are spreading and mostly unfurled, (vii) leaves are fully emerged and unfurled (**Supplementary Figure S1**). Leaf bud burst date was defined as the date at which greater or equal to 50% of the buds per tree reached stage six of development.

Flower phenology was monitored in 2014 from 1st March. From the date of first flower opening, trees were examined every 3 days and number of buds, flowers, and senescent flowers were recorded according to the following definitions adapted from Wagner et al. (2014); (i) budding, petals are clearly visible but not distinctly unfurled (ii) flowering, when the corolla is separated sufficient for four distinct petals and the stamen of the flower to be clearly identified, (iii) senescent, flower shows clear signs of senescence such as petal loss, discolouration and wilting. The day at which 50% of buds per tree were flowering/had flowered was then identified.

#### Pathogen Incidence

Tree health was monitored over the duration of the experiment and a pathogen was observed in July 2014 which naturally infected all of the study trees at approximately the same time. *Neonectria ditissima* (apple canker) is a highly infectious pathogen which disperses readily by spores produced continually throughout the year. No significant effect of tree position upon pathogen incidence was detected demonstrating even distribution of pathogen spores with the pattern of infection in our experimental orchard matching that which occurs in commercial orchards (Beresford and Kim, 2010; Weber, 2014). Infection of apple tissue samples with the fungal pathogen *N. ditissima* was confirmed by sequencing four representative fungal fruiting bodies from infected branches. Genomic DNA was extracted using methods described elsewhere (Gardes and Bruns, 1993) with a purification step using GeneClean (QbioGene). The fungal ITS1-5.8S-ITS2 region was amplified and sequenced using primers ITS1F and ITS4 (White et al., 1990; Gardes and Bruns, 1993) using PicoMaxx (Stratagene) and BigDye Terminator v.3.1 (Thermo Fisher Scientific) with a 3730 DNA Analyzer (Applied Biosystems) (GenBank accession numbers MG679892-5). The DNA sequences were compared to the NCBI database using the BLAST search algorithm, and all had ≥98% similarity over ≥99% of query coverage to a sequence of *N. ditissima* (accession no. JK7355309.1). DNA sequencing, and regular checks throughout the experiment, did not detect any other pathogens. In accordance with agricultural management practises infected branches were removed during July 2014 and 2015; we used the total length of infected material removed per tree as an indicator of pathogen damage. This is a standard quantitative measure of disease severity which has been used by other recent studies of *Neonectria ditissima* infection of apple (Garkava-Gustavsson et al., 2013; Gómez-Cortecero et al., 2016).

### Statistical Analyses

All analyses were performed in R studio version 3.3.1 (R Core Team, 2017); we conducted separate analyses for each year. We present the results of models that include all two-way interaction terms for all main effects, but not higher level interactions as preliminary analyses revealed that these were almost invariably non-significant (*P* < 0.05 in just seven out of 117 cases) and were never consistently significant across years (higher level interactions were only significant in one of the 3 years).

We conducted four-way ANOVAs using the ‘lme4’ package to test the effect of mycorrhizal status (inoculated or not), nutrient status (high or low), scion type (Michelin or Dabinett) and rootstock (MM106 or MM111) upon all tree nutrient status parameters (leaf tissue P, N, C concentration, C:N ratio, chlorophyll content), tree growth [height, trunk diameter, above and below ground biomass and root length (which was log_10_ transformed)], productivity (fruit and flower production) and disease resistance. All two-way interactions between the four main effects (AMF, nutrient input, scion, and rootstock treatments) were included and block was incorporated as a random factor.

We conducted a three-way ANOVA to test the effect of nutrient status, scion and rootstock type upon AMF colonisation and included all two-way interactions between main effects (AMF inoculation was excluded as a main effect from this analysis as no non-inoculated trees were colonised).

We modelled leaf and flower phenology as functions of mycorrhizal status, nutrient status, scion type and rootstock using Generalised Linear Models constructed with the ‘nlme’ package and Poisson error distribution. All two-way interactions between predictors were included.

## Results

### Mycorrhizal Colonisation

**FIGURE 1:**
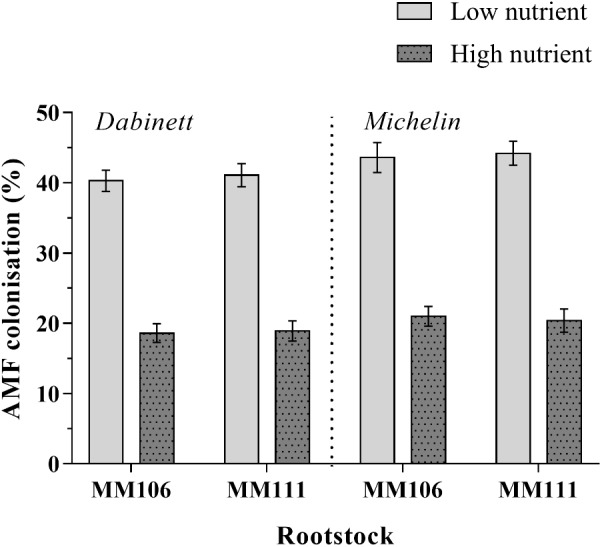
Root colonisation of apple trees (*Malus pumila*) inoculated with two species of arbuscular mycorrhizal fungi (AMF). Mean AMF colonisation after 3 years of growth under high and low nutrient treatments and for both Dabinett and Michelin scions is presented. Error bars represent ± SE per treatment (*n* = 20). AMF colonisation was significantly affected by both scion type (*P* < 0.05) and nutrient treatment (*P* < 0.001) but not rootstock type.

All inoculated trees were colonised by AMF (range of colonisation rates 11–54%) whilst all non-inoculated trees remained free of AMF. For inoculated trees, high nutrient treatment resulted in a significant 53% reduction in AMF colonisation compared to low nutrient treatment (**Tables 1**, **2** and **Figure 1**). The AMF colonisation was also affected by scion type; trees with Michelin scions on average showed an 8% higher colonisation compared to those with Dabinett scions (**Tables 1**, **2** and **Figure 1**). No difference between colonisation of rootstocks was found (**Tables 1**, **2**).

**Table 1:** Main factor effects and significant two-way interactions from four-way ANOVA analyses of apple (*Malus pumila*) performance metrics from a 3-year growth experiment; the four main factor effects tested are (1) arbuscular mycorrhizal fungal (AMF) inoculation (AMF inoculation or non-inoculation), (2) nutrient addition (low or high), (3) scion type (Dabinett or Michelin), and (4) rootstock type (MM106 or MM111).

Performance metric	Year		AMF inoculation		Nutrient		Scion		Rootstock		AMF inoculation × Rootstock		Nutrient × Rootstock		Nutrient × Scion		Rootstock × Scion	
										
		df	*F*	*P*	*F*	*P*	*F*	*P*	*F*	*P*	*F*	*P*	*F*	*P*	*F*	*P*	*F*	*P*
AMF colonisation (%)	3	1,72	–	-	**399.454**	**<0.001**	**5.197**	**0.025**	0.059	0.808	–	–	–	–	–	–	–	–
Leaf P (mg g^−1^)	1	1,148	1.210	0.273	0.233	0.630	0.481	0.489	0.001	0.974	–	–	–	–	–	–	–	–
	2	1,148	0.436	0.510	1.593	0.208	1.373	0.243	0.141	0.707	–	–	–	–	**7.028**	**0.008**	–	–
	3	1,148	2.568	0.111	**111.058**	**<0.001**	3.023	0.084	1.691	0.195	–	–	–	–	–	–	–	–
Leaf N (mg g^−1^)	1	1,148	1.031	0.312	1.241	0.267	0.737	0.392	2.602	0.109	–	–	–	–	–	–	–	–
	2	1,148	0.495	0.483	2.894	0.091	3.589	0.060	**8.343**	**0.004**	**5.297**	**0.022**	–	–	–	–	–	–
	3	1,148	0.001	0.978	**232.944**	**<0.001**	**4.518**	**0.035**	**17.164**	**<0.001**	–	–	–	–	–	–	–	–
Leaf C (mg g^−1^)	1	1,148	3.501	0.063	0.165	0.684	1.419	0.235	0.072	0.788	–	–	–	–	–	–	–	–
	2	1,148	0.072	0.789	0.180	0.671	**8.915**	**0.003**	**5.664**	**0.018**	–	–	–	–	–	–	–	–
	3	1,148	1.058	0.305	**10.704**	**0.001**	1.762	0.186	0.425	0.515	–	–	–	–	–	–	–	–
Leaf C:N	1	1,148	0.932	0.335	1.748	0.188	1.483	0.225	3.071	0.081	–	–	–	–	–	–	–	–
	2	1,148	0.286	0.593	2.924	0.089	0.649	0.421	**10.269**	**0.001**	**5.602**	**0.019**	–	–	–	–	–	–
	3	1,148	0.600	0.439	**242.327**	**<0.001**	**5.052**	**0.026**	**19.945**	**<0.001**	–	–	–	–	–	–	–	–
Leaf chlorophyll (mg g^−1^)	1	1,148	1.403	0.238	1.277	0.260	**89.805**	**<0.001**	**12.759**	**<0.001**	–	–	–	–	–	–	**5.273**	**0.023**
	2	1,148	0.025	0.873	2.751	0.099	**8.825**	**0.003**	**4.526**	**0.035**	–	–	–	–	–	–	**11.701**	**<0.001**
	3	1,148	0.646	0.423	**95.412**	**<0.001**	**92.116**	**<0.001**	**31.019**	**<0.001**	–	–	–	–	–	–	**23.899**	**<0.001**
Tree height (cm)	1	1,148	1.988	0.160	1.129	0.289	**72.387**	**<0.001**	**5.478**	**0.020**	–	–	–	–	–	–	**6.217**	**0.013**
	2	1,148	**6.553**	**0.011**	0.532	0.467	**12.138**	**<0.001**	**12.600**	**<0.001**	–	–	–	–	–	–	**4.921**	**0.028**
	3	1,148	0.684	0.409	0.405	0.525	**65.449**	**<0.001**	**27.164**	**<0.001**	–	–	–	–	–	–	–	–
Trunk diameter (cm)	1	1,148	0.039	0.844	2.173	0.142	**29.218**	**<0.001**	0.241	0.623	–	–	–	–	–	–	**9.891**	**0.002**
	2	1,148	0.005	0.944	0.398	0.529	**41.614**	**<0.001**	**20.138**	**<0.001**	–	–	–	–	–	–	**25.48**	**<0.001**
	3	1,148	0.144	0.705	1.023	0.313	**20.138**	**<0.001**	**14.381**	**<0.001**	–	–	–	–	–	–	**11.221**	**0.001**
Dry shoot biomass (g)	3	1,36	0.699	0.408	0.025	0.875	1.454	0.235	**6.788**	**0.013**	–	–	**4.974**	**0.032**	–	–	–	–
Dry root biomass (g)	3	1,36	1.010	0.321	**7.696**	**0.008**	**8.379**	**0.006**	**15.842**	**<0.001**	–	–	–	–	–	–	–	–
Root length (cm)	3	1,148	**5.958**	**0.015**	0.288	0.592	**8.669**	**0.003**	0.260	0.611	–	–	**7.235**	**0.007**	–	–	–	–
Fruit biomass (g)	1	1,148	**4.439**	**0.036**	0.144	0.705	**47.251**	**<0.001**	**14.176**	**<0.001**	–	–	–	–	–	–	–	–
Fruit number	1	1,148	2.378	0.125	0.013	0.910	**37.579**	**<0.001**	**8.386**	**0.004**	–	–	–	–	–	–	–	–
Flower number	2	1,148	0.056	0.813	**6.388**	**0.012**	**42.871**	**<0.001**	**54.712**	**<0.001**	–	–	–	–	–	–	–	–
	3	1,148	2.603	0.108	0.001	0.996	0.896	0.345	**11.989**	**<0.001**	–	–	–	–	–	–	–	–
Pathogen infection (cm)	2 and 3	1,148	**10.811**	**0.001**	0.573	0.450	**27.249**	**<0.001**	**14.736**	**<0.001**	–	–	–	–	–	–	**4.971**	**0.027**

*Significant results (P < 0.05) are in bold.*

**Table 2:** Treatment means and ± SE for metrics of apple (*Malus pumila*) performance from a three year growth experiment; percentage differences between the two levels of each of the four treatments (1) arbuscular mycorrhizal fungal (AMF) inoculation (AMF inoculation or non-inoculation), (2) fertiliser addition (low or high), (3) scion type (Dabinett or Michelin), and (4) rootstock type (MM106 or MM111), are also presented.

Performance metric	Year	AMF: Non-inoculation		AMF: Inoculation		% change	Nutrient: Low		Nutrient: High		% change	Scion: Dabinett		Scion: Michelin		% change	Rootstock: MM106		Rootstock: MM111		% change
													
		Mean	SE	Mean	SE		Mean	SE	Mean	SE		Mean	SE	Mean	SE		Mean	SE	Mean	SE	
AMF colonisation (%)	3	–	–	31.02	1.39	–	**42.30**	**0.88**	**19.73**	**0.71**	**-53.36**	**29.73**	**1.89**	**32.30**	**2.03**	**8.64**	30.88	1.95	31.15	2.00	0.87
Leaf P (mg g^−1^)	1	2.95	0.10	3.10	0.10	5.08	2.99	0.10	3.06	0.10	2.34	2.98	0.10	3.07	0.09	3.02	3.03	0.10	3.02	0.01	-0.33
	2	2.23	0.05	2.18	0.05	-2.24	2.16	0.04	2.25	0.05	4.16	2.17	0.05	2.25	0.05	3.68	2.22	0.05	2.19	0.05	-1.35
	3	2.28	0.08	2.44	0.09	7.01	**1.85**	**0.05**	**2.87**	**0.08**	**55.13**	2.45	0.09	2.28	0.08	-6.93	2.30	0.09	2.43	0.08	5.65
Leaf N (mg g^−1^)	1	27.50	0.26	27.13	0.33	-1.33	27.11	0.32	27.51	0.27	1.48	27.16	0.31	27.47	0.29	1.14	27.02	0.30	27.60	0.29	2.16
	2	22.62	0.25	22.63	0.30	0.04	22.19	0.26	23.06	0.28	3.92	22.92	0.32	22.34	0.23	-2.53	**22.25**	**0.23**	**23.01**	**0.31**	**3.41**
	3	21.53	0.37	21.52	0.42	-0.04	**18.85**	**0.24**	**24.20**	**0.28**	**28.38**	**21.89**	**0.41**	**21.15**	**0.38**	–**3.40**	**20.79**	**0.39**	**22.25**	**0.39**	**6.99**
Leaf C (mg g^−1^)	1	469.31	0.97	466.68	1.01	-0.50	467.71	1.04	468.28	0.96	0.121	468.83	1.05	467.16	0.94	-0.35	468.18	0.99	467.81	1.01	-0.07
	2	467.24	1.18	466.82	1.05	-0.08	467.36	0.91	466.70	1.30	-0.14	**469.33**	**1.09**	**464.73**	**1.08**	–**0.98**	**468.86**	**1.01**	**465.20**	**1.18**	–**0.78**
	3	476.13	1.30	477.33	1.35	0.252	**479.58**	**1.30**	**474.33**	**1.28**	–**1.09**	475.89	1.33	478.02	1.31	0.44	477.48	1.38	476.43	1.26	-0.21
Leaf C:N ratio	1	17.18	0.15	17.40	0.21	0.28	17.44	0.20	17.14	0.16	-1.72	17.43	0.19	17.15	0.18	-1.60	17.49	0.19	17.09	0.18	-2.28
	2	20.97	0.24	20.79	0.27	-0.85	21.18	0.25	20.58	0.25	-2.83	20.74	0.29	21.02	0.21	1.35	**21.44**	**0.25**	**20.32**	**0.25**	–**5.22**
	3	22.65	0.40	22.96	0.49	1.36	**25.79**	**0.35**	**19.82**	**0.24**	–**23.14**	**22.37**	**0.44**	**23.23**	**0.45**	**3.84**	**23.66**	**0.48**	**21.95**	**0.40**	–**7.22**
Leaf chlorophyll (mg g^−1^)	1	2.92	0.12	2.94	0.01	0.58	2.94	0.01	2.92	0.01	-0.50	**2.87**	**0.01**	**3.00**	**0.01**	**4.52**	**2.96**	**0.01**	**2.91**	**0.01**	–**1.65**
	2	2.57	0.01	2.58	0.01	0.11	2.59	0.01	2.56	0.01	-0.84	**2.55**	**0.01**	**2.59**	**0.009**	**1.56**	**2.59**	**0.01**	**2.56**	**0.01**	–**1.07**
	3	2.47	0.01	2.46	0.01	-0.44	**2.40**	**0.01**	**2.53**	**0.01**	**5.36**	**2.40**	**0.01**	**2.53**	**0.01**	**5.27**	**2.50**	**0.01**	**2.43**	**0.01**	–**2.91**
Tree height (cm)	1	196.20	1.42	193.70	1.69	-1.29	195.90	1.53	194.00	1.60	-0.96	**187.30**	**1.28**	**202.60**	**1.34**	**8.16**	**192.80**	**1.42**	**197.10**	**1.67**	**2.23**
	2	**179.10**	**4.26**	**193.70**	**4.36**	**8.15**	188.50	4.34	184.40	4.42	-2.17	**176.50**	**4.07**	**196.40**	**4.41**	**11.27**	**196.60**	**4.60**	**179.30**	**3.8**	–**10.32**
	3	171.10	3.38	174.50	3.92	1.98	174.10	3.86	171.55	3.45	-1.49	**156.40**	**3.03**	**189.20**	**3.30**	**20.97**	**183.40**	**3.70**	**162.50**	**3.22**	–**11.39**
Trunk diameter (cm)	1	1.86	0.03	1.85	0.03	-0.26	1.84	0.03	1.87	0.03	2.00	**1.79**	**0.01**	**1.92**	**0.03**	**7.64**	1.86	0.02	1.85	0.03	-0.69
	2	2.30	0.03	2.31	0.03	0.12	2.29	0.03	2.32	0.03	1.00	**2.19**	**0.01**	**2.42**	**0.03**	**10.47**	**2.23**	**0.02**	**2.39**	**0.03**	**7.17**
	3	2.59	0.01	2.58	0.02	-0.57	2.56	0.02	2.60	0.01	1.55	**2.49**	**0.01**	**2.67**	**0.02**	**7.12**	**2.51**	**0.02**	**2.66**	**0.02**	**5.97**
Dry shoot biomass (g)	3	526.83	22.23	501.89	23.55	-4.73	516.73	23.66	512.00	22.41	-0.91	496.37	19.63	532.36	25.47	7.25	**553.24**	**22.49**	**475.49**	**20.62**	–**14.05**
Dry root biomass (g)	3	323.75	20.11	302.91	20.58	-6.43	**342.08**	**20.99**	**284.58**	**18.03**	–**16.80**	**283.33**	**14.76**	**343.33**	**23.27**	**21.17**	**354.58**	**18.32**	**272.08**	**18.81**	–**23.26**
Root length (cm)	3	**119.60**	**7.28**	**96.47**	**5.71**	–**19.33**	106.0	6.28	110.1	7.04	3.86	**97.21**	**6.69**	**118.9**	**6.43**	**22.31**	110.0	6.81	106.1	6.25	-3.64
Fruit biomass (g)	1	**133.66**	**23.13**	**85.59**	**15.68**	–**35.00**	113.95	22.03	105.30	17.60	-7.59	**88.04**	**23.20**	**31.21**	**10.08**	–**83.40**	**152.58**	**23.30**	**66.68**	**14.36**	–**56.29**
Fruit number	1	1.80	0.31	1.28	0.22	-28.88	1.56	0.28	1.52	0.25	-2.56	**2.56**	**0.29**	**0.52**	**0.18**	–**79.60**	**2.02**	**0.30**	**1.06**	**0.23**	–**47.50**
Flower number	2	32.55	5.49	33.88	5.47	4.08	26.07	4.46	40.36	6.24	54.81	**51.72**	**6.06**	**14.71**	**3.84**	–**71.55**	**54.12**	**6.27**	**12.31**	**3.11**	–**77.25**
	3	7.75	1.73	13.23	2.45	70.70	10.47	2.11	10.51	2.17	0.38	11.85	2.13	9.13	2.14	-22.95	**15.51**	**2.60**	**5.47**	**1.33**	–**64.73**
Pathogen infection (cm)	2 and 3	**570.11**	**26.09**	**465.50**	**25.02**	–**18.34**	505.76	26.86	529.85	25.52	4.76	**600.85**	**23.03**	**434.76**	**25.90**	–**27.64**	**456.73**	**27.36**	**578.87**	**23.08**	**26.74**

*Significant results (P < 0.05) from four-way ANOVA analyses are in bold (see **Table 1** for full details).*

### Tree Nutrient Status

#### Leaf Phosphorus

No significant effect of AMF inoculation or rootstock type upon leaf P concentration was shown. In year three only, trees receiving high nutrient application showed a significant increase in leaf P concentration (**Tables 1**, **2**). A significant interaction between nutrient and scion treatment was found for year two only (**Table 1**) where trees with Michelin scions had increased leaf P under high compared to low nutrient treatment, however, the opposite was shown for Dabinett scions (**Supplementary Table S3**).

#### Leaf Nitrogen

Trees receiving high nutrient treatment showed on average higher leaf N across all years of the experiment, however, this increase was only significant in year three (**Tables 1**, **2**). For both year one and two, trees with MM111 rootstocks showed significantly greater concentration of leaf tissue N than those with MM106 rootstocks (**Tables 1**, **2**) and an interaction between mycorrhiza and rootstock was shown in year two (**Table 1** and **Supplementary Table S4**). Trees with MM111 rootstocks showed increased leaf N when inoculated, however, inoculation reduced leaf N of those with MM106 rootstocks relative to non-inoculated trees (**Supplementary Table S4**). In year three also, Dabinett scions showed increased leaf N compared to Michelin scions (**Tables 1**, **2**).

#### Leaf Carbon and C:N Ratio

In the third year of the experiment trees receiving high nutrient treatment showed both a significantly reduced leaf tissue C content and C:N ratio compared to low nutrient trees (**Tables 1**, **2**). Significant differences in leaf C and C:N were shown between rootstock and scions during year two and three of the experiment although these effects were not consistent between years (**Tables 1**, **2**). A significant interaction between the effect of AMF inoculation and rootstock was shown for C:N ratio in year two (**Table 1**) where inoculation reduced C:N ratio of trees with MM111 rootstocks but increased C:N ratio of those with MM106 rootstocks (**Supplementary Table S4**). This is likely be driven by the significant interaction between rootstock and AMF inoculation observed for leaf N in year two as no interaction between these factors was shown for leaf C.

#### Leaf Chlorophyll

For year three only, trees receiving high nutrient application showed a significant increase in mean leaf chlorophyll compared to trees receiving low nutrient treatment (**Tables 1**, **2**). Tree rootstock and scion type had a significant effect upon leaf chlorophyll across all years of the experiment with Michelin scions and MM106 rootstock showing on average the highest chlorophyll content (**Tables 1**, **2**). A significant interaction between the effect of scion and rootstock upon leaf chlorophyll was found for all 3 years of the experiment (**Table 1**) with Dabinett scions on MM111 rootstocks showing the lowest chlorophyll content for both year two and three (**Supplementary Table S5**).

### Tree Growth

#### Height and Diameter

For year two only, AMF inoculated trees showed a significant increase in height compared to non-inoculated trees (**Tables 1**, **2**) but inoculation had no effect on trunk diameter. Nutrient treatment did not affect tree height or trunk diameter in any year (**Tables 1**, **2**).

Trees with Michelin scions were consistently taller throughout the experiment (**Tables 1**, **2**); however, the effect on rootstock on tree height differed between years. Trees with MM111 rootstocks were significantly taller in year one (**Tables 1**, **2**) and a significant interaction was shown between rootstock and scion type; height of Michelin scions increased when grown on MM111 compared to MM106 rootstocks, however, rootstock type caused negligible difference in height of Dabinett scions (**Supplementary Table S5**). In contrast, trees with MM111 rootstock were significantly shorter in year two and three compared to MM106 (**Tables 1**, **2**). This may partly be explained by increased susceptibility of trees with MM111 rootstocks to apple canker which became established in year two leading to removal of material from infected trees and thus height reduction (see “Disease Resistance” section of Results). Similarly, Dabinett MM111 trees which were most susceptible to canker damage compared to other rootstock scion combinations, showed substantial reduction in height during year two, driving a significant interaction between rootstock and scion for year two tree height (**Supplementary Table S5**).

Trees with Michelin scions had on average larger trunk diameters in year one. There was also a significant interaction between scion and rootstock for trunk diameter with MM106 rootstocks increasing trunk diameter for Dabinett trees but reducing trunk diameter of Michelin trees relative to MM111 rootstocks (**Supplementary Table S5**). For year two and three trunk diameter was affected by both scion and rootstock type (**Tables 1**, **2**). This was driven by a significant interaction between rootstock and scion for both years whereby trunk diameter of Michelin MM111 trees was consistently much larger than the other rootstock scion combinations (**Supplementary Table S5**).

#### Biomass

**FIGURE 2:**
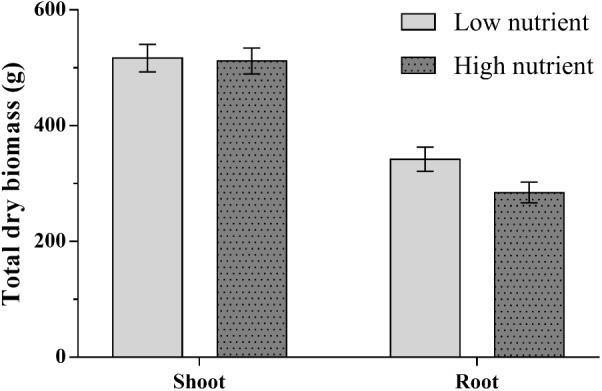
Mean total dry above and below ground biomass for apple trees (*Malus pumila*) grown under high and low nutrient treatments. Error bars represent ± SE per treatment (*n* = 24). Below ground biomass was significantly affected by nutrient treatment (*P* < 0.01), scion type (*P* < 0.01), and rootstock type (*P* < 0.001). Above ground biomass was significantly affected by rootstock type (*P* < 0.05) only.

Root biomass was significantly reduced in trees receiving high nutrient application (**Figure 2** and **Tables 1**, **2**). Scion and rootstock type also significantly affected root biomass; on average larger root systems were found for trees with Michelin scions and MM106 rootstocks (**Tables 1**, **2**). A significant interaction was shown between nutrient treatment and rootstock for above ground biomass; high nutrient treatment increased above ground biomass of trees with MM106 rootstocks but decreased above ground biomass of those with MM111 rootstocks relative to low nutrient treatment (**Table 2** and **Supplementary Table S6**).

#### Root Length

Mycorrhizal inoculation reduced root length compared to non-inoculated trees (**Tables 1**, **2**). As AMF inoculated trees did not show reduced root biomass this implies that AMF trees had a coarser root system than those which were not colonised. Trees with Dabinett scions showed significantly greater root length (**Tables 1**, **2**) despite a lower total root biomass indicating a finer root system than trees with Michelin scions. A significant interaction between the effect of nutrient treatment and rootstock was shown (**Table 1**) with high nutrient treatment increasing root length of MM106 rootstocks but reducing root length of MM111 rootstocks compared to root length under low nutrient conditions (**Supplementary Table S6**).

#### Leaf and Flower Phenology

No significant treatment effects upon leaf bud burst or flowering dates were shown. On average trees came into leaf after 60 days in year two (60th day = 29th April 2014) and 77 days in year three (77th day = 16th May 2015) reflecting the colder spring temperatures of 2015 (data not shown).

### Tree Productivity

#### Fruit and Flower Production

The first year’s fruit crop was low as expected for newly planted trees (Williams, 1996). Non-inoculated trees yielded a significantly greater fruit biomass compared to those that were inoculated (**Tables 1**, **2**); however, the total number of fruit produced per tree was not affected by inoculation indicating that fruit biomass rather than fruit number was reduced in the AMF treatment. First year fruit biomass and number was also significantly affected by both tree scion and rootstock with Dabinett scions and MM106 rootstocks yielding a greater number and biomass of fruit (**Tables 1**, **2**).

Mycorrhizal inoculation did not influence flower production (**Tables 1**, **2**). Trees with MM106 rootstocks produced a significantly greater number of flowers in both years two and three. Year two flower production was significantly increased for trees receiving high nutrient treatment and trees with Dabinett scions (**Tables 1**, **2**).

### Disease Resistance

**FIGURE 3:**
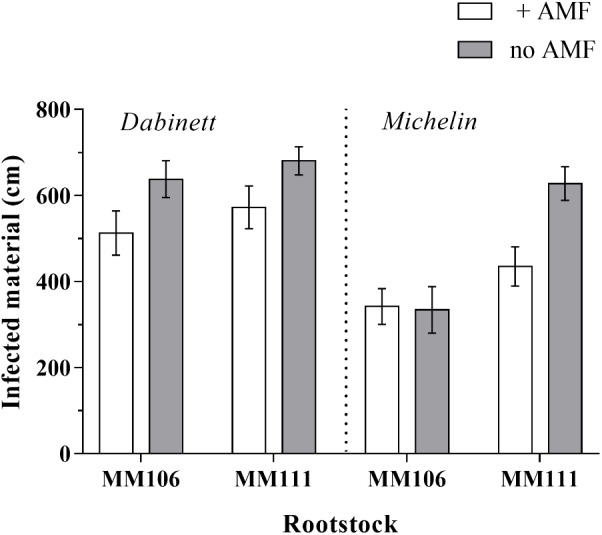
Total apple tree (*Malus pumila*) material infected by the fungal pathogen *Neonectria ditissima*. Trees were grown with and without inoculation with arbuscular mycorrhizal fungi (AMF). Error bars represent ± SE per treatment (*n* = 20). Total pathogen infected material was significantly affected by AMF inoculation (*P* = 0.001), scion type (*P* < 0.001) and rootstock type (*P* < 0.001).

The AMF inoculation significantly reduced the amount of plant material infected by the fungal pathogen *N. ditissima* by an average of 18% compared to non-inoculated trees (**Figure 3** and **Tables 1**, **2**). Large differences in pathogen susceptibility were found between both scion and rootstock types with significantly lower canker incidence for Michelin scions and MM106 rootstocks (**Tables 1**, **2**). There was also a significant interaction between the effect of rootstock and scion type upon disease resistance (**Figure 3** and **Table 1**) with canker incidence of rootstock/scion combinations substantially reduced on Michelin MM106 trees compared to other scion rootstock combinations (**Figure 3** and **Supplementary Table S5**).

## Discussion

We provide a robust, controlled and fully factorial experiment that quantifies the impacts of AMF colonisation in a woody perennial crop on multiple performance indicators including plant growth, nutritional status, cropping potential and disease resistance. Our findings shed light on how fertiliser applications influence AMF colonisation, AMF-induced disease resistance and the impact of above ground genotypes on rootstock associations with AMF.

### Effects of Fertiliser Applications

Best practise fertiliser application guidelines for newly planted young apple trees significantly reduced AMF colonisation rates. Similar reductions in AMF colonisation of arable crops by inorganic fertiliser applications have been widely reported (Kahiluoto et al., 2001; Gosling et al., 2006; Li et al., 2006). This may be explained by the ‘functional equilibrium model’ (Johnson, 2010; Johnson et al., 2013) whereby plants invest more energy into plant and fungal structures which optimise capture of the resources (i.e., carbon, nitrogen, and phosphorus) most limited in availability, therefore reducing carbon allocation to AMF under none nutrient limited conditions. However, whilst fertiliser application rate did not affect leaf nutrient status in year one, by year three both leaf P and N were significantly reduced in low nutrient trees (N by year two) irrespective of mycorrhizal inoculation treatment suggesting that increased colonisation of low nutrient trees by AMF did not compensate for reduced fertiliser inputs. The ‘functional equilibrium model’ may also explain why standard fertiliser application rates reduced root biomass (Poorter et al., 2012), a metric that is positively associated with drought resistance (Brunner et al., 2015). Growers may thus benefit from limiting nutrient applications during the early stages of orchard establishment due to reduced costs of inputs, potentially improved drought resistance and increased AMF colonisation rates (and associated benefits) without adverse impacts on plant health. Reduced fertiliser applications will also deliver a wide range of environmental benefits (Tilman et al., 2001).

### Mycorrhizal Effects on Disease Resistance

Inoculation with mycorrhizal fungi improved resistance of apple trees to the fungal pathogen *N. ditissima* reducing, on average, the amount of infected material by 18%. There is variation, however, in the impact of AMF on canker resistance with negligible effects for Michelin trees grown on MM106 rootstock. To our knowledge, this is the first study to report the benefit of mycorrhizas for resistance to this pathogen which infects apple orchards globally and can severely reduce productivity of canker sensitive varieties, which generate a large proportion of global apple production (Weber, 2014). This extends previous work suggesting that AMF can increase apple seedlings’ resistance to pathogens (Utkhede and Smith, 2000; Raj and Sharma, 2009; Krishna et al., 2010) to commercially relevant settings, as the apple industry relies on grafts rather than growth of seedlings. Furthermore, the two AMF taxa we used for inoculation (*R. irregularis* and *F. mosseae*) are commercially available, enhancing the industrial relevance of our research, and have previously been shown to increase resistance to pathogens in other crop species (Liu et al., 2007; Song et al., 2011; Hao et al., 2012).

Although the mechanisms are not fully understood, AMF may affect host plant disease resistance through several non-mutually exclusive pathways: (i) alleviating environmental stress such as drought (Augé, 2004; Bowles et al., 2016), (ii) improving host plant nutrition and subsequent health (Smith and Smith, 2011; Cavagnaro, 2014; Bowles et al., 2017a), (iii) by priming the plant immune system (Jung et al., 2012), and (iv) through synergistic interactions with plant growth-promoting rhizobacteria (Cameron et al., 2013; Pérez-de-Luque et al., 2017). Our experimental trees were supplied with supplementary water and (with the exception of significantly increased leaf N and corresponding reduced C:N ratios for AMF inoculated trees with MM111 rootstocks during year two) we found no evidence that AMF colonisation increased N and P leaf nutrient concentrations or changed C:N ratios. These findings thus rule out the first two hypotheses for how AMF enhanced disease resistance. Colonisation by AMF may elicit a priming response whereby the plant immune system is pre-conditioned so that although defences are not actively expressed, when under attack the defence system is able to respond more rapidly and to a greater extent than a plant which is not primed, thus improving defensive capacity (Jung et al., 2012). Colonisation by AMF may also indirectly influence plant defence by altering metabolite exudation from roots and thus influencing recruitment of plant growth-promoting rhizobacteria in the rhizosphere (Frey-Klett et al., 2007; Cameron et al., 2013) and thus affect systemic plant disease resistance (Lioussanne, 2013; D’Alessandro et al., 2014). We therefore speculate that that AMF induced defence response and/or indirect effects upon rhizosphere microbial communities may explain increased resistance of apple trees to *N. ditissima* observed in this study. However, further experiments are required to confirm this. Irrespective of the mechanism, fertiliser induced reductions in mycorrhizal colonisation (from 40 to 20%) did not alter the magnitude of pathogen suppression, suggesting that even relatively low levels of mycorrhizal colonisation are able to influence plant defence systems.

### Mycorrhizal Effects on Growth Rates, Nutrient Status, Phenology, and Yield

We hypothesised that AMF would positively influence tree nutrient status and consequently growth rates. However, we found that AMF inoculation did not influence leaf C:N ratios, N, P and chlorophyll concentrations, plant height, trunk diameter or above ground biomass in trees growing under conditions that otherwise were similar to those of commercial apple orchards. AMF can be more efficient than roots at nutrient capture due to their ability to respond rapidly to nutrient availability and the finer diameter of their hyphae compared to roots allowing them greater access to soil pores and greater efficiency at exploring larger areas of soil (Drew et al., 2003; Cavagnaro et al., 2005). The last of these mechanisms could not occur in our experiment due to its pot based system, perhaps partly explaining the lack of effect of AMF on nutrient status. Alternatively, lack of enhanced tissue nutrient levels in AMF trees could be explained by down-regulation of plant nutrient uptake in response to AMF colonisation generating no net nutritional gain to AMF host plants (Smith et al., 2004; Nagy et al., 2009).

There is also variation in the ability of specific AMF taxa to deliver nutritional benefits (Forge et al., 2001; Zhu et al., 2001), with previous work (Maherali and Klironomos, 2007) finding limited nutritional benefits of *R. irregularis* and *F. mosseae* compared to other AMF taxa. It is thus plausible that the lack of nutritional benefits to apple trees of AMF inoculation is influenced by the design of our experimental system. It may, however, also partly arise from a more general mechanism of reduced investment in fine root growth, i.e., the root type responsible for nutrient capture, in plants colonised by AMF. Indeed, our results showed decreased root length of AMF trees compared to non-AMF trees despite no differences in overall root biomass suggesting that colonised trees shift allocation in root growth from fine roots to coarser roots.

We investigated the effect of AMF upon leaf and flower phenology based upon recent work which has suggested that microbial communities, including AMF, have a role in determining plant flowering time through potential effects upon phenotypic plasticity of flower phenology (Wagner et al., 2014; Vaingankar and Rodrigues, 2015; Liu et al., 2017). Timing of leaf and flower development is of agronomical importance due to potential effects on growth and flower production and consequently fruit yield. In particular, flowering date is critical as climate conditions and pollinator activity during this period determine flower set and the annual fruit crop (Williams, 1996). Our data showed no difference between mycorrhiza-inoculated and non-inoculated trees in the timing of leaf or flower phenology – although effects of AMF upon flower phenology can vary between crop species and AMF taxa (Philip et al., 2001; Liu et al., 2017). The fruit crop from all trees following the first growing season was very low (averaging approximately one fruit per tree), this is expected given the tree age (Williams, 1996). Mycorrhizal trees showed significantly reduced yield in the first year of the experiment (35% reduction) compared to non-inoculated trees. One explanation for this could be the carbon cost of AMF symbiosis, i.e., the resource allocation hypothesis, with AMF acquiring carbon that would otherwise be allocated to fruit production (Guo et al., 1994). It was not possible to measure fruit yield for the subsequent 2 years of the experiment due to very low fruit production as a result of pathogen infection. However, measurement of flower production in the second and third years showed no difference between inoculated and non-inoculated trees suggesting that yield potential was similar.

### Rootstock and Scion Effects

Previous research with herbaceous species has suggested that crop breeding in agricultural soils with high nutrient levels and low levels of AMF activity may have impaired the ability of subsequent germplasm to form mycorrhizal associations (Hetrick et al., 1993, 1996). Rootstock type (MM106 and MM111) did not affect mycorrhizal colonisation. Whilst colonisation results are not always a good predictor of mycorrhizal activity (Hetrick et al., 1993; Zhu et al., 2001), it is noteworthy that these two widely planted commercial rootstocks are able to achieve high levels of colonisation (up to 43%) despite being subject to intensive artificial selection pressure. Remarkably, AMF colonisation was influenced more by scion type than rootstock, with greater colonisation of roots grown with Michelin scions than Dabinett scions. This matches results of recent work on *Citrus* (Song et al., 2015). Root exudates can influence AMF colonisation (Broeckling et al., 2008; Kiers et al., 2011), and our work suggests that some of these exudates may be synthesised in the scion (thus explaining differences in colonisation between scion types). Further research into how interactions between above and below ground plant tissue alter AMF symbioses is required to ensure that AMF symbiotic potential is maximised in current breeding programmes.

### Synthesis and Future Directions

We find that currently advocated fertiliser regimes reduce AMF colonisation rates of apple trees, and have negligible nutritional or growth benefits during at least the first two growing seasons. Standard fertiliser application rates also appear to reduce root biomass potentially increasing adverse impacts of drought stress. Most significantly, we find strong evidence that AMF colonisation by *R. irregularis* and *F. mosseae* can significantly reduce the intensity of infection by *N. ditissima* which causes apple canker – a major pathogen. Furthermore we show that the genetic identity of above ground plant tissue has stronger impacts on AMF colonisation than rootstock genotypes, drawing attention to an important and overlooked focus for plant breeding programmes that seek to maximise mycorrhizal status of grafted crops, and the associated benefits that can be delivered by AMF. This study emphasises the need to further understand the role of AMF in plant protection against pathogens and highlights the potential for AMF within sustainable agriculture.

## Author Contributions

DB, DC, and KE designed the study. MB performed the molecular identification of pathogen samples. DB performed the research, data analysis, and wrote the first draft of the manuscript. TC, TD, DC, and KE contributed with data interpretation and wrote sections of the manuscript. All authors contributed to manuscript revision, read, and approved the final submitted version.

## Conflict of Interest Statement

The authors declare that the research was conducted in the absence of any commercial or financial relationships that could be construed as a potential conflict of interest.

## Supplementary Material

FIGURE S1 The seven stages used for scoring apple (*Malus pumila*) leaf phenology.

TABLE S1 Apple tree macronutrient applications.

TABLE S2 Apple tree micronutrient applications.

TABLE S3 Treatment means and ± SE for parameters of apple (*Malus pumila*) performance per nutrient treatment (low or high) and scion type (Dabinett or Michelin), where significant interactions between nutrient treatment and scion type were reported by four-way ANOVA analysis.

TABLE S4 Treatment means and ± SE for parameters of apple (*Malus pumila*) performance per arbuscular mycorrhizal fungal (AMF) inoculation treatment (AMF inoculation or non-inoculation) and rootstock type (MM106 or MM111), where significant interactions between inoculation treatment and rootstock type were reported by four-way ANOVA analysis.

TABLE S5 Treatment means and ± SE for parameters of apple (*Malus pumila*) performance per scion (Dabinett or Michelin) and rootstock type (MM106 or MM111), where significant interactions between scion and rootstock type were reported by four-way ANOVA analysis.

TABLE S6 Treatment means and ± SE for parameters of apple (*Malus pumila*) performance per nutrient treatment (low or high) and rootstock type (MM106 or MM111), where significant interactions between nutrient treatment and rootstock type were reported by four-way ANOVA analysis.

NOTES S1 Summary of fertiliser application treatment.

## References

[B1] Albacete A. Martinez-Andujar C. Martinez-Perez A. Thompson A. J. Dodd I. C. Perez-Alfocea F. (2015). Unravelling rootstock-scion interactions to improve food security. *J. Exp. Bot.* 66 2211–2226. 10.1093/jxb/erv027 25754404 PMC4986720

[B2] Allen S. E. (1989). *Chemical Analysis of Ecological Materials.* Oxford: Blackwell Scientific.

[B3] An Z. Q. Shen T. Wang H. G. (1993). Mycorrhizal fungi in relation to growth and mineral nutrition of apple seedlings. *Sci. Hortic.* 54 275–285. 10.1016/0304-4238(93)90106-Z

[B4] Arnon D. I. (1949). Copper enzymes in isolated chloroplasts. Polyphenoloxidase in *Beta vulgaris. Plant Physiol.* 24 1–15. 10.1104/pp.24.1.1 16654194 PMC437905

[B5] Augé R. M. (2004). Arbuscular mycorrhizae and soil/plant water relations. *Can. J. Soil Sci.* 84 373–381. 10.4141/S04-002

[B6] Bauer S. E. Tsigaridis K. Miller R. (2016). Significant atmospheric aerosol pollution caused by world food cultivation. *Geophys. Res. Lett.* 43 5394–5400. 10.1002/2016GL068354

[B7] Bender S. F. Conen F. van der Heijden M. G. A. (2015). Mycorrhizal effects on nutrient cycling, nutrient leaching and N2O production in experimental grassland. *Soil Biol. Biochem.* 80 283–292. 10.1016/j.soilbio.2014.10.016

[B8] Beresford R. M. Kim K. S. (2010). Identification of regional climatic conditions favourable for development of european canker of apple. *Phytopathology* 101 135–146. 10.1094/PHYTO-05-10-0137 20795854

[B9] Bowles T. M. Barrios-Masias F. H. Carlisle E. A. Cavagnaro T. R. Jackson L. E. (2016). Effects of arbuscular mycorrhizae on tomato yield, nutrient uptake, water relations, and soil carbon dynamics under deficit irrigation in field conditions. *Sci. Total Environ.* 566–567, 1223–1234. 10.1016/j.scitotenv.2016.05.178 27266519

[B10] Bowles T. M. Jackson L. E. Cavagnaro T. R. (2017a). Mycorrhizal fungi enhance plant nutrient acquisition and modulate nitrogen loss with variable water regimes. *Glob. Change Biol.* 24 171–182. 10.1111/gcb.13884 28862782

[B11] Bowles T. M. Jackson L. E. Loeher M. Cavagnaro T. R. (2017b). Ecological intensification and arbuscular mycorrhizas: a meta-analysis of tillage and cover crop effects. *J. Appl. Ecol.* 54 1785–1793. 10.1111/1365-2664.12815

[B12] Broeckling C. D. Broz A. K. Bergelson J. Manter D. K. Vivanco J. M. (2008). Root exudates regulate soil fungal community composition and diversity. *Appl. Environ. Microbiol.* 74 738–744. 10.1128/AEM.02188-07 18083870 PMC2227741

[B13] Brundrett M. Peterson L. Melville L. Addy H. McGonigle T. Schaffer G. (1994). *Practical Methods in Mycorrhizal Research.* Toronto ON: Mycologue Publications.

[B14] Brunner I. Herzog C. Dawes M. A. Arend M. Sperisen C. (2015). How tree roots respond to drought. *Front. Plant Sci.* 6:547. 10.3389/fpls.2015.00547 26284083 PMC4518277

[B15] Cameron D. D. (2010). Arbuscular mycorrhizal fungi as (agro)ecosystem engineers. *Plant Soil* 333 1–5. 10.1007/s11104-010-0361-y

[B16] Cameron D. D. Neal A. L. van Wees S. C. M. Ton J. (2013). Mycorrhiza-induced resistance: more than the sum of its parts? *Trends Plant Sci.* 18 539–545. 10.1016/j.tplants.2013.06.004 23871659 PMC4194313

[B17] Cameron D. D. Preiss K. Gebauer G. Read D. J. (2009). The chlorophyll-containing orchid *Corallorhiza trifida* derives little carbon through photosynthesis. *New Phytol.* 183 358–364. 10.1111/j.1469-8137.2009.02853.x 19402877

[B18] Cavagnaro T. R. (2014). Impacts of compost application on the formation and functioning of arbuscular mycorrhizas. *Soil Biol. Biochem.* 78 38–44. 10.1016/j.soilbio.2014.07.007

[B19] Cavagnaro T. R. Bender S. F. Asghari H. R. van der Heijden M. G. A. (2015). The role of arbuscular mycorrhizas in reducing soil nutrient loss. *Trends Plant Sci.* 20 283–290. 10.1016/j.tplants.2015.03.004 25840500

[B20] Cavagnaro T. R. Smith F. A. Smith S. E. Jakobsen I. (2005). Functional diversity in arbuscular mycorrhizas: exploitation of soil patches with different phosphate enrichment differs among fungal species. *Plant Cell Environ.* 28 642–650. 10.1111/j.1365-3040.2005.01310.x

[B21] Cooke L. R. (1999). The influence of fungicide sprays on infection of Apple cv. Bramley’s Seedling by *Nectria galligena*. *Eur. J. Plant Pathol.* 105 783–790. 10.1023/A:1008778900607

[B22] Cordell D. Drangert J. O. White S. (2009). The story of phosphorus: global food security and food for thought. *Glob. Environ. Change* 19 292–305. 10.1016/j.gloenvcha.2008.10.009

[B23] Cordell D. White S. (2014). “Life’s bottleneck: sustaining the world’s phosphorus for a food secure future,” in *Annual Review of Environment and Resources*, eds Gadgil A. Liverman D. M. (Palo Alto, CA: Annual Reviews), 161–188.

[B24] Daei G. Ardekani M. R. Rejali F. Teimuri S. Miransari M. (2009). Alleviation of salinity stress on wheat yield, yield components, and nutrient uptake using arbuscular mycorrhizal fungi under field conditions. *J. Plant Physiol.* 166 617–625. 10.1016/j.jplph.2008.09.013 19100656

[B25] D’Alessandro M. Erb M. Ton J. Brandenburg A. Karlen D. Zopfi J. (2014). Volatiles produced by soil-borne endophytic bacteria increase plant pathogen resistance and affect tritrophic interactions. *Plant Cell Environ.* 37 813–826. 10.1111/pce.12220 24127750 PMC4194311

[B26] DEFRA (2010). *Fertiliser Manual (RB209)*, 8th Edn. Norwich: The Stationary Office.

[B27] Drew E. A. Murray R. S. Smith S. E. Jakobsen I. (2003). Beyond the rhizosphere: growth and function of arbuscular mycorrhizal external hyphae in sands of varying pore sizes. *Plant Soil* 251 105–114. 10.1023/A:1022932414788

[B28] Elser J. Bennett E. (2011). Phosphorus cycle: a broken biogeochemical cycle. *Nature* 478 29–31. 10.1038/478029a 21979027

[B29] FAO (2018). *FAOSTAT Database Collections. Food and Agriculture Organization of the United Nations.* Available at: http://www.fao.org/faostat/en/#data/QC [accessed 1 9 2018].

[B30] Fester T. Sawers R. (2011). Progress and challenges in agricultural applications of arbuscular mycorrhizal fungi. *Crit. Rev. Plant Sci.* 30 459–470. 10.1080/07352689.2011.605741

[B31] Foley J. A. Ramankutty N. Brauman K. A. Cassidy E. S. Gerber J. S. Johnston M. (2011). Solutions for a cultivated planet. *Nature* 478 337–342. 10.1038/nature10452 21993620

[B32] Forge T. Muehlchen A. Hackenberg C. Neilsen G. Vrain T. (2001). Effects of preplant inoculation of apple (*Malus domestica* Borkh.) with arbuscular mycorrhizal fungi on population growth of the root-lesion nematode, Pratylenchus penetrans. *Plant Soil* 236 185–196. 10.1023/A:1012743028974

[B33] Frey-Klett P. Garbaye J. Tarkka M. (2007). The mycorrhiza helper bacteria revisited. *New Phytol.* 176 22–36. 10.1111/j.1469-8137.2007.02191.x 17803639

[B34] Gange A. C. Brown V. K. Aplin D. M. (2005). Ecological specificity of arbuscular mycorrhizae: evidence from foliar- and seed-feeding insects. *Ecology* 86 603–611. 10.1890/04-0967

[B35] Gardes M. Bruns T. D. (1993). ITS primers with enhanced specificity for basidiomycetes - application to the identification of mycorrhizae and rusts. *Mol. Ecol.* 2 113–118. 10.1111/j.1365-294X.1993.tb00005.x 8180733

[B36] Garkava-Gustavsson L. Zborowska A. Sehic J. Rur M. Nybom H. Englund J. E. (2013). Screening of apple cultivars for resistance to European canker, *Neonectria ditissima*. *Acta Hortic.* 976 529–536. 10.17660/ActaHortic.2013.976.75

[B37] Gómez-Cortecero A. Saville R. J. Scheper R. W. A. Bowen J. K. Agripino De Medeiros H. Kingsnorth J. (2016). Variation in host and pathogen in the *Neonectria/Malus* Interaction; toward an understanding of the genetic basis of resistance to European canker. *Front. Plant Sci.* 7:1365. 10.3389/fpls.2016.01365 27695463 PMC5023678

[B38] Gosling P. Hodge A. Goodlass G. Bending G. D. (2006). Arbuscular mycorrhizal fungi and organic farming. *Agric. Ecosyst. Environ.* 113 17–35. 10.1016/j.agee.2005.09.009

[B39] Guo B. Z. An Z. Q. Hendrix J. W. (1994). A mycorrhizal pathogen (*Glomus macrocarpum* Tul. & Tul.) of tobacco: effects of long- and short-term cropping on the mycorrhizal fungal community and stunt disease. *Appl. Soil Ecol.* 1 269–276. 10.1016/0929-1393(94)90004-3

[B40] Hao Z. Fayolle L. Tuinen D. van Chatagnier O. Li X. Gianinazzi S. (2012). Local and systemic mycorrhiza-induced protection against the ectoparasitic nematode *Xiphinema index* involves priming of defence gene responses in grapevine. *J. Exp. Bot.* 63 3657–3672. 10.1093/jxb/ers046 22407649 PMC3388824

[B41] Herrera-Estrella L. López-Arredondo D. (2016). Phosphorus: The underrated element for feeding the world. *Trends Plant Sci.* 21 461–463. 10.1016/j.tplants.2016.04.010 27160806

[B42] Hetrick B. Wilson G. Cox T. (1993). Mycorrhizal dependence of modern wheat cultivars and ancestors - a synthesis. *Can. J. Bot.* 71 512–518. 10.1139/b93-056

[B43] Hetrick B. A. D. Wilson G. W. T. Todd T. C. (1996). Mycorrhizal response in wheat cultivars: Relationship to phosphorus. *Can. J. Bot.* 74 19–25. 10.1139/b96-003

[B44] Horton P. (2017). We need radical change in how we produce and consume food. *Food Secur.* 9 1323–1327. 10.1007/s12571-017-0740-9

[B45] Johnson N. C. (2010). Resource stoichiometry elucidates the structure and function of arbuscular mycorrhizas across scales. *New Phytol.* 185 631–647. 10.1111/j.1469-8137.2009.03110.x 19968797

[B46] Johnson N. C. Angelard C. Sanders I. R. Kiers E. T. (2013). Predicting community and ecosystem outcomes of mycorrhizal responses to global change. *Ecol. Lett.* 16 140–153. 10.1111/ele.12085 23679013

[B47] Joosten F. J. Dijkxhoorn Y. Sertse Y. Ruben R. (2015). *How does the Fruit and Vegetable Sector Contribute to Food and Nutrition Security?.* The Hague: LEI Wageningen UR.

[B48] Jung S. C. Martinez-Medina A. Lopez-Raez J. A. Pozo M. J. (2012). Mycorrhiza-induced resistance and priming of plant defenses. *J. Chem. Ecol.* 38 651–664. 10.1007/s10886-012-0134-6 22623151

[B49] Kahiluoto H. Ketoja E. Vestberg M. Saarela I. (2001). Promotion of AM utilization through reduced P fertilization 2. Field studies. *Plant Soil* 231 65–79. 10.1023/A:1010366400009

[B50] Kiers E. T. Duhamel M. Beesetty Y. Mensah J. A. Franken O. Verbruggen E. (2011). Reciprocal rewards stabilize cooperation in the mycorrhizal symbiosis. *Science* 333 880–882. 10.1126/science.1208473 21836016

[B51] Köhl L. van der Heijden M. G. A. (2016). Arbuscular mycorrhizal fungal species differ in their effect on nutrient leaching. *Soil Biol. Biochem.* 94 191–199. 10.1016/j.soilbio.2015.11.019

[B52] Krishna H. Das B. Attri B. L. Grover M. Ahmed N. (2010). Suppression of *Botryosphaeria* canker of apple by arbuscular mycorrhizal fungi. *Crop Prot.* 29 1049–1054. 10.1016/j.cropro.2010.05.005

[B53] Leff J. W. Lynch R. C. Kane N. C. Fierer N. (2017). Plant domestication and the assembly of bacterial and fungal communities associated with strains of the common sunflower, *Helianthus annuus*. *New Phytol.* 214 412–423. 10.1111/nph.14323 27879004

[B54] Li H. Smith S. E. Holloway R. E. Zhu Y. Smith F. A. (2006). Arbuscular mycorrhizal fungi contribute to phosphorus uptake by wheat grown in a phosphorus-fixing soil even in the absence of positive growth responses. *New Phytol.* 172 536–543. 10.1111/j.1469-8137.2006.01846.x 17083683

[B55] Lioussanne L. (2013). The role of the arbuscular mycorrhiza-associated rhizobacteria in the biocontrol of soilborne phytopathogens: a review. *Span. J. Agric. Res.* 8 51–61. 10.5424/sjar/201008S1-5301

[B56] Liu J. Y. Maldonado-Mendoza I. Lopez-Meyer M. Cheung F. Town C. D. Harrison M. J. (2007). Arbuscular mycorrhizal symbiosis is accompanied by local and systemic alterations in gene expression and an increase in disease resistance in the shoots. *Plant J.* 50 529–544. 10.1111/j.1365-313X.2007.03069.x 17419842

[B57] Liu S. Guo H. Xu J. Song Z. Song S. Tang J. (2017). Arbuscular mycorrhizal fungi differ in affecting the flowering of a host plant under two soil phosphorus conditions. *J. Plant Ecol.* 11 623–631. 10.1093/jpe/rtx038

[B58] Mader P. Fliessbach A. Dubois D. Gunst L. Fried P. Niggli U. (2002). Soil fertility and biodiversity in organic farming. *Science* 296 1694–1697. 10.1126/science.1071148 12040197

[B59] Maherali M. Klironomos J. N. (2007). Influence of phylogeny on fungal community assembly and ecosystem functioning. *Science* 316 1746–1748. 10.1126/science.1143082 17588930

[B60] Matsubara Y. Karikomi T. Ikuta M. Hori H. Ishikawa S. Harada T. (1996). Effect of arbuscular Mycorrhizal fungus inoculation on growth of apple (*Malus* ssp) seedlings. *J. Jpn. Soc. Hortic. Sci.* 65 297–302. 10.2503/jjshs.65.297

[B61] McCracken A. R. Berrie A. Barbara D. J. Locke T. Cooke L. R. Phelps K. (2003). Relative significance of nursery infections and orchard inoculum in the development and spread of apple canker (*Nectria galligena*) in young orchards. *Plant Pathol.* 52 553–566. 10.1046/j.1365-3059.2003.00924.x

[B62] McGonigle T. Miller M. Evans D. Fairchild G. Swan J. (1990). A new method which gives an objective measure of colonization of roots. *New Phytol.* 115 495–501. 10.1111/j.1469-8137.1990.tb00476.x 33874272

[B63] Miller D. Domoto P. Walker C. (1985). Mycorrhizal fungi at 18 apple rootstock plantings in the united-states. *New Phytol.* 100 379–391. 10.1111/j.1469-8137.1985.tb02787.x

[B64] Nagy R. Drissner D. Amrhein N. Jakobsen I. Bucher M. (2009). Mycorrhizal phosphate uptake pathway in tomato is phosphorus-repressible and transcriptionally regulated. *New Phytol.* 181 950–959. 10.1111/j.1469-8137.2008.02721.x 19140941

[B65] Öpik M. Moora M. Liira J. Zobel M. (2006). Composition of root-colonizing arbuscular mycorrhizal fungal communities in different ecosystems around the Globe. *J. Ecol.* 94 778–790. 10.1111/j.1365-2745.2006.01136.x

[B66] Pérez-de-Luque A. Tille S. Johnson I. Pascual-Pardo D. Cameron D. D. (2017). The interactive effects of arbuscular mycorrhiza and plant growth-promoting rhizobacteria synergistically enhance host plant defences against pathogens. *Sci. Rep.* 7:6409. 10.1038/s41598-017-16697-4 29180695 PMC5703727

[B67] Pérez-Jaramillo J. E. Mendes R. Raaijmakers J. M. (2016). Impact of plant domestication on rhizosphere microbiome assembly and functions. *Plant Mol. Biol.* 90 635–644. 10.1007/s11103-015-0337-7 26085172 PMC4819786

[B68] Philip L. J. Posluszny U. Klironomos J. N. (2001). The influence of mycorrhizal colonization on the vegetative growth and sexual reproductive potential of *Lythrum salicaria* L. *Can. J. Bot.* 79 381–388. 10.1139/b01-010

[B69] Poorter H. Niklas K. J. Reich P. B. Oleksyn J. Poot P. Mommer L. (2012). Biomass allocation to leaves, stems and roots: meta-analyses of interspecific variation and environmental control. *New Phytol.* 193 30–50. 10.1111/j.1469-8137.2011.03952.x 22085245

[B70] Pretty J. Sutherland W. J. Ashby J. Auburn J. Baulcombe D. Bell M. (2010). The top 100 questions of importance to the future of global agriculture. *Int. J. Agric. Sustain.* 8 219–236. 10.3763/ijas.2010.0534

[B71] R Core Team (2017). *R: A Language and Environment for Statistical Computing.* Vienna: R Foundation for Statistical Computing.

[B72] Raj H. Sharma S. D. (2009). Integration of soil solarization and chemical sterilization with beneficial microorganisms for the control of white root rot and growth of nursery apple. *Sci. Hortic.* 119 126–131. 10.1016/j.scienta.2008.07.025

[B73] Rillig M. C. Mummey D. L. (2006). Mycorrhizas and soil structure. *New Phytol.* 171 41–53. 10.1111/j.1469-8137.2006.01750.x 16771981

[B74] Royer M. Larbat R. Le Bot J. Adamowicz S. Robin C. (2013). Is the C:N ratio a reliable indicator of C allocation to primary and defence-related metabolisms in tomato? *Phytochemistry* 88 25–33. 10.1016/j.phytochem.2012.12.003 23312460

[B75] Schneider C. A. Rasband W. S. Eliceiri K. W. (2012). NIH Image to ImageJ: 25 years of image analysis. *Nat. Methods* 9 671–675. 10.1038/nmeth.2089 22930834 PMC5554542

[B76] Smith S. E. Read D. (2008). *Mycorrhizal Symbiosis.* Cambridge: Academic Press.

[B77] Smith S. E. Smith F. A. (2011). “Roles of arbuscular Mycorrhizas in plant nutrition and growth: new paradigms from cellular to ecosystem scales,” in *Annual Review of Plant Biology*, eds Merchant S. S. Briggs W. R. Ort D. (Palo Alto, CA: Annual Reviews), 227–250. 10.1146/annurev-arplant-042110-103846 21391813

[B78] Smith S. E. Smith F. A. Jakobsen I. (2004). Functional diversity in arbuscular mycorrhizal (AM) symbioses: the contribution of the mycorrhizal P uptake pathway is not correlated with mycorrhizal responses in growth or total P uptake. *New Phytol.* 162 511–524. 10.1111/j.1469-8137.2004.01039.x

[B79] Song F. Pan Z. Bai F. An J. Liu J. Guo W. (2015). The scion/rootstock genotypes and habitats affect arbuscular mycorrhizal fungal community in citrus. *Front. Microbiol.* 6:1372. 10.3389/fmicb.2015.01372 26648932 PMC4664953

[B80] Song Y. Y. Cao M. Xie L. J. Liang X. T. Zeng R. S. Su Y. J. (2011). Induction of DIMBOA accumulation and systemic defence responses as a mechanism of enhanced resistance of mycorrhizal corn (*Zea mays* L.) to sheath blight. *Mycorrhiza* 21 721–731. 10.1007/s00572-011-0380-4 21484338

[B81] Thirkell T. J. Charters M. D. Elliott A. J. Sait S. M. Field K. J. (2017). Are mycorrhizal fungi our sustainable saviours? Considerations for achieving food security. *J. Ecol.* 105 921–929. 10.1111/1365-2745.12788

[B82] Tilman D. Balzer C. Hill J. Befort B. L. (2011). Global food demand and the sustainable intensification of agriculture. *Proc. Natl. Acad. Sci. U.S.A.* 108 20260–20264. 10.1073/pnas.1116437108 22106295 PMC3250154

[B83] Tilman D. Clark M. Williams D. R. Kimmel K. Polasky S. Packer C. (2017). Future threats to biodiversity and pathways to their prevention. *Nature* 546 73–81. 10.1038/nature22900 28569796

[B84] Tilman D. Fargione J. Wolff B. D’Antonio C. Dobson A. Howarth R. (2001). Forecasting agriculturally driven global environmental change. *Science* 292 281–284. 10.1126/science.1057544 11303102

[B85] Tubiello F. N. Salvatore M. Ferrara A. F. House J. Federici S. Rossi S. (2015). The contribution of agriculture, forestry and other land use activities to global warming, 1990–2012. *Glob. Change Biol.* 21 2655–2660. 10.1111/gcb.12865 25580828

[B86] Utkhede R. S. Smith E. M. (2000). Impact of chemical, biological and cultural treatments on the growth and yield of apple in replant-disease soil. *Austral. Plant Pathol.* 29 129–136. 10.1071/AP00021

[B87] Vaingankar J. D. Rodrigues B. F. (2015). Effect of arbuscular mycorrhizal (AM) inoculation on growth and flowering in *Crossandra infundibuliformis* (L.) Nees. *J. Plant Nutr.* 38 1478–1488. 10.1080/01904167.2014.957398

[B88] Varga S. Kytöviita M. M. (2010). Gender dimorphism and mycorrhizal symbiosis affect floral visitors and reproductive output in *Geranium sylvaticum*. *Funct. Ecol.* 24 750–758. 10.1111/j.1365-2435.2010.01708.x

[B89] Veiga R. S. L. Jansa J. Frossard E. van der Heijden M. G. A. (2011). Can arbuscular mycorrhizal fungi reduce the growth of agricultural weeds? *PLoS One* 6:e27825. 10.1371/journal.pone.0027825 22164216 PMC3229497

[B90] Wagner M. R. Lundberg D. S. Coleman-Derr D. Tringe S. G. Dangl J. L. Mitchell-Olds T. (2014). Natural soil microbes alter flowering phenology and the intensity of selection on flowering time in a wild *Arabidopsis* relative. *Ecol. Lett.* 17 717–726. 10.1111/ele.12276 24698177 PMC4048358

[B91] Weber R. W. S. (2014). Biology and control of the apple canker fungus *Neonectria ditissima* (syn. N. galligena) from a Northwestern European perspective. *Erwerbs Obstbau* 56 95–107. 10.1007/s10341-014-0210-x

[B92] White T. Bruns T. Lee S. Taylor J. (1990). “Amplification and direct sequencing of fungal ribosomal RNA genes for phylogenetics,” in *PCR Protocols: A Guide to Methods and Applications PCR Protocols: A Guide to Methods and Applications*, eds Innis M. A. Gelfand D. H. Sninsky J. J. White T. J. (Cambridge, MA: Academic Press), 315–322.

[B93] Williams R. (1996). *Cider and Juice Apples: Growing and Processing.* Bristol: University of Bristol Printing Unit.

[B94] Wilson G. W. T. Rice C. W. Rillig M. C. Springer A. Hartnett D. C. (2009). Soil aggregation and carbon sequestration are tightly correlated with the abundance of arbuscular mycorrhizal fungi: results from long-term field experiments. *Ecol. Lett.* 12 452–461. 10.1111/j.1461-0248.2009.01303.x 19320689

[B95] Zhu Y. G. Smith S. E. Barritt A. R. Smith F. A. (2001). Phosphorus (P) efficiencies and mycorrhizal responsiveness of old and modern wheat cultivars. *Plant Soil* 237 249–255. 10.1023/A:1013343811110

