# Supplementary material for: The Effects of Arbuscular Mycorrhizal Fungal Colonisation on Nutrient Status, Growth, Productivity, and Canker Resistance of Apple (Malus pumila)
Source: Front Microbiol. 2018 Jul 3;9:1461. doi: 10.3389/fmicb.2018.01461 (PMC6037770; doi:10.3389/fmicb.2018.01461)
Supplement: FIGURE S1 — The seven stages used for scoring apple (Malus pumila) leaf phenology. [file Data_Sheet_1.DOC]

Supplementary Material

Arbuscular mycorrhizal fungi reduce susceptibility of apple (*Malus pumila*) to biotic stress

**Despina Berdeni^*^, T. E. Anne Cotton, Tim J. Daniell, Martin I. Bidartondo, Duncan D. Cameron and Karl L. Evans**

*** Correspondence:** Dr Despina Berdeni: despina.berdeni@sheffield.ac.uk

The following Supporting Information is available for this article:

**Fig. S1** The seven stages used for scoring apple (*Malus pumila*) leaf phenology.

**Table S1** Apple tree macronutrient applications.

**Table S2** Apple tree micronutrient applications.

**Table S3** Treatment means and ± SE for parameters of apple (*Malus pumila*) performance per nutrient treatment (low or high) and scion type (Dabinett or Michelin), where significant interactions between nutrient treatment and scion type were reported by four-way ANOVA analysis.

**Table S4** Treatment means and ± SE for parameters of apple (*Malus pumila*) performance per arbuscular mycorrhizal fungal (AMF) inoculation treatment (AMF inoculation or non-inoculation) and rootstock type (MM106 or MM111), where significant interactions between inoculation treatment and rootstock type were reported by four-way ANOVA analysis.

**Table S5** Treatment means and ± SE for parameters of apple (*Malus pumila*) performance per scion (Dabinett or Michelin) and rootstock type (MM106 or MM111), where significant interactions between scion and rootstock type were reported by four-way ANOVA analysis.

**Table S6** Treatment means and ± SE for parameters of apple (*Malus pumila*) performance per nutrient treatment (low or high) and rootstock type (MM106 or MM111), where significant interactions between nutrient treatment and rootstock type were reported by four-way ANOVA analysis.

**Notes S1** Summary of fertiliser application treatment.


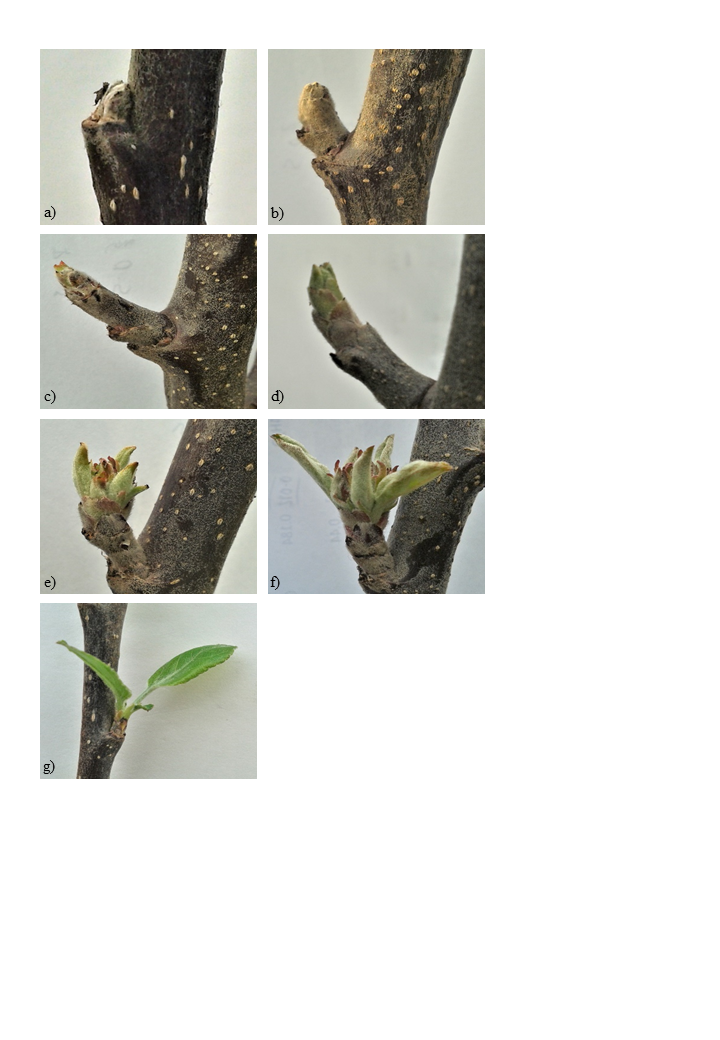


**Fig. 1** The seven stages used for scoring apple (*Malus pumila*) leaf phenology: (a) leaf bud dormant, (b) bud swollen – heavily swollen but no sign of opening, (c) bud beginning to open but less than half is green, (d) over half of the bud is green but leaf tips point inward, (e) leaf tips point outward and leaf unfurling is clear, (f) leaves are spreading and mostly unfurled, (g) leaves are fully emerged and unfurled.

**Table S1** Apple tree macronutrient applications.

| Nutrient | DEFRA advised annual application (g per tree) | Total nutrient application per tree (g yr^-1^) | | Application per tree (g) applied per 2 weeks of the growing season | |
| --- | --- | --- | --- | --- | --- |
|  |  | High nutrient | Low nutrient | High nutrient | Low nutrient |
| N | 93.00 | 23.25 | 2.32 | 2.32 | 0.23 |
| P | 85.30 | 21.32 | 2.13 | 2.13 | 0.21 |
| K | 166.00 | 41.51 | 4.15 | 4.15 | 0.41 |
| Mg | 165.00 | 41.25 | 4.12 | 4.12 | 0.41 |

**Table S2** Apple tree micronutrient applications

| Nutrient | Total application (ug year ^-1^) | Application per 2 weeks of the growing season (µg) | Nutrient form |
| --- | --- | --- | --- |
| Fe | 152.15 | 15.21 | FeEDTA |
| Mn | 25.46 | 2.54 | MnSO_4_.4H_2_O |
| B | 20.02 | 2.00 | H_3_BO_3_ |
| Mo | 5.59 | 0.55 | (NH_4_)6Mo_7_O_2_.4H_2_O |
| Zn | 4.00 | 0.40 | ZnS0_4_.7H_2_0 |
| Cu | 4.00 | 0.40 | CuSO_4_.5H_2_0 |
| S | 18.84 | 1.88 | MnSO_4_.4H_2_O |

**Table S3** Treatment means and ± SE for parameters of apple (*Malus pumila*) performance per nutrient treatment (low or high) and scion type (Dabinett or Michelin), where significant interactions between nutrient treatment and scion type were reported by four-way ANOVA analysis.

| Parameter | Year | Low nutrient | | | | High nutrient | | | |
| --- | --- | --- | --- | --- | --- | --- | --- | --- | --- |
|  |  | Dabinett | | Michelin | | Dabinett | | Michelin | |
|  |  | Mean | SE | Mean | SE | Mean | SE | Mean | SE |
| Leaf P (mg g^-1^) | 2 | 2.22 | 0.07 | 2.11 | 0.05 | 2.12 | 0.06 | 2.39 | 0.08 |

**Table S4** Treatment means and ± SE for parameters of apple (*Malus pumila*) performance per arbuscular mycorrhizal fungal (AMF) inoculation treatment (AMF inoculation or non-inoculation) and rootstock type (MM106 or MM111), where significant interactions between inoculation treatment and rootstock type were reported by four-way ANOVA analysis.

| Parameter | Year | AMF inoculation | | | | Non-inoculation | | | |
| --- | --- | --- | --- | --- | --- | --- | --- | --- | --- |
|  |  | MM111 | | MM106 | | MM111 | | MM106 | |
|  |  | Mean | SE | Mean | SE | Mean | SE | Mean | SE |
| Leaf N (mg g^-1^) | 2 | 23.72 | 0.42 | 21.78 | 0.36 | 22.60 | 0.39 | 22.38 | 0.31 |
| Leaf C:N | 2 | 19.82 | 3.13 | 21.76 | 3.44 | 20.83 | 3.29 | 21.13 | 3.34 |

**Table S5** Treatment means and ± SE for parameters of apple (*Malus pumila*) performance per scion (Dabinett or Michelin) and rootstock type (MM106 or MM111), where significant interactions between scion and rootstock type were reported by four-way ANOVA analysis.

| Parameter | Year | Dabinett | | | | Michelin | | | |
| --- | --- | --- | --- | --- | --- | --- | --- | --- | --- |
|  |  | MM106 | | MM111 | | MM106 | | MM111 | |
|  |  | Mean | SE | Mean | SE | Mean | SE | Mean | SE |
| Chlorophyll (mg g^-1^) | 1 | 2.91 | 0.01 | 2.99 | 0.01 | 3.01 | 0.01 | 2.99 | 0.008 |
|  | 2 | 2.59 | 0.01 | 2.52 | 0.01 | 2.59 | 0.01 | 2.60 | 0.01 |
|  | 3 | 2.47 | 0.01 | 2.33 | 0.01 | 2.53 | 0.01 | 2.52 | 0.01 |
| Height (cm) | 1 | 187.45 | 1.47 | 187.20 | 2.12 | 198.30 | 2.14 | 207.00 | 1.33 |
|  | 2 | 193.00 | 6.45 | 160.07 | 3.40 | 200.22 | 6.62 | 192.62 | 5.86 |
| Trunk Diameter (cm) | 1 | 1.83 | 0.02 | 1.74 | 0.02 | 1.89 | 0.03 | 1.96 | 0.02 |
|  | 2 | 2.20 | 0.02 | 2.18 | 0.02 | 2.25 | 0.03 | 2.59 | 0.05 |
|  | 3 | 2.49 | 0.02 | 2.50 | 0.02 | 2.53 | 0.04 | 2.81 | 0.05 |
| Pathogen infection (cm) | 2 & 3 | 575.25 | 34.43 | 626.45 | 30.50 | 338.22 | 33.59 | 531.30 | 33.36 |

**Table S6** Treatment means and ± SE for parameters of apple (*Malus pumila*) performance per nutrient treatment (low or high) and rootstock type (MM106 or MM111), where significant interactions between nutrient treatment and rootstock type were reported by four-way ANOVA analysis.

|  |  | Low nutrient | | | | High nutrient | | | |
| --- | --- | --- | --- | --- | --- | --- | --- | --- | --- |
| Parameter | Year | MM111 | | MM106 | | MM106 | | MM111 | |
|  |  | Mean | SE | Mean | SE | Mean | SE | Mean | SE |
| Dry shoot biomass (g) | 3 | 522.32 | 18.61 | 511.13 | 18.82 | 439.85 | 10.49 | 584.15 | 15.36 |
| Root length (cm) | 3 | 98.07 | 8.92 | 113.89 | 8.77 | 98.35 | 9.61 | 121.89 | 10.07 |

**Notes S1** Summary of fertiliser application treatment.

Nutrient applications were based on the DEFRA recommendations for cider apple orchards in the first year following planting for a soil index of 0. Application rates per tree were calculated assuming that trees were planted at a density of 750 trees per hectare following industry recommendations for the planting of the selected varieties and rootstocks within a commercial bush orchard. This was then reduced to 1/4^th^ to account for the improved efficiency of tree nutrient uptake due to growth in pots. Trees in the ‘low nutrient’ treatment received 1/10^th^ of the macronutrient applications applied to the ‘high nutrient’ treatment. All nutrients were applied in the fertiliser forms as used for a commercial bush orchard: Yara Krista MgS (16% MgO), Yara Krista Plus K (13.7% N, 46.3% K_2_O), Yara Krista MAP (12% N, 61% P_2_O_5_). To reduce leaching losses which would be expected to be higher from pots than from field soils, total nutrient application was divided into treatments applied every 2 weeks for 20 weeks over the growing season (May to mid-September). All trees were applied with the same micronutrients in solution (Fe, Mn, S, B, Mo, Zn and Cu).
